# Investigating the Allelopathic and Bioherbicidal Potential of *Solidago altissima* with a Focus on Chemical Signaling in *Trifolium repens*

**DOI:** 10.3390/plants14010096

**Published:** 2024-12-31

**Authors:** Ho-Jun Gam, Arjun Adhikari, Yosep Kang, Md. Injamum-Ul-Hoque, Shifa Shaffique, Ji-In Woo, Jin Ryeol Jeon, Byeong-Kwan An, Min Young Back, Ki-Yong Kim, Sang-Mo Kang, In-Jung Lee

**Affiliations:** 1 Department of Applied Biosciences, Kyungpook National University, Daegu 41566, Republic of Korea; 2023001144@knu.ac.kr (H.-J.G.); arjun@knu.ac.kr (A.A.); alfm4545@knu.ac.kr (Y.K.); mdinjamum92@knu.ac.kr (M.I.-U.-H.); shifa.2021@knu.ac.kr (S.S.); wjxsj99@knu.ac.kr (J.-I.W.); 98micael@knu.ac.kr (J.R.J.); abkabk1020@knu.ac.kr (B.-K.A.); miny4310@knu.ac.kr (M.Y.B.); sangmo@knu.ac.kr (S.-M.K.); 2 National Institute of Animal Science, Rural Development Administration (RDA), Cheonan 31000, Republic of Korea; kimky77@korea.kr; 3 Institute of Agricultural Science and Technology, Kyungpook National University, Daegu 41566, Republic of Korea

**Keywords:** phytotoxicity, weeds, ROS, antioxidants, phytohormone, methyl kolavenate

## Abstract

Invasive weed species exhibit both advantages, such as the potential for allelochemicals in bioherbicide development, and risks, including their threat to crop production. Therefore, this study aims to identify an allelochemical from *Solidago altissima*, an invasive weed species. The dose-dependent effects of *S. altissima* shoot and root extracts (SSE, SRE) on the signaling in the forage crop *Trifolium repens* and germination in various weed species (*Echinochloa oryzicola*, *Cyperus microiria*, *Alopecurus aequalis*, *Portulaca oleracea*, and *Amaranthus retroflexus*) were evaluated. The results showed that the *T. repens* seedlings treated with root extracts exhibited a significant decrease in plant height, dry weight, and chlorophyll content, along with an increase in H_2_O_2_ levels. Additionally, antioxidant activities, such as superoxide dismutase, catalase, and peroxidase enzyme activities, were significantly elevated in *T. repens* treated with SRE. Moreover, SRE treatment significantly inhibited the seed germination of all tested weed species in a concentration-dependent manner. Gas chromatography-mass spectrometry analysis of *S. altissima* root extract identified a high concentration of methyl kolavenate, a clerodane diterpene predicted to act as a phytotoxic agent. These findings highlight the potential of *S. altissima* for the development of crop-protective agents while emphasizing its potential risks in agriculture.

## 1. Introduction

The spread of invasive weed species (WS) is considered a significant threat to sustainable agriculture owing to its potential to cause ecosystem disruption, biodiversity reduction, and lead to economic losses [1]. Among these invasive species, *Solidago altissima* L., commonly known as goldenrod (Common name; tall goldenrod, family; *Asteraceae*), of the Asteraceae family, is classified as a perennial weed. Originally native to North America, this species is widely recognized as a significant threat in Europe and Asia [2]. *S. altissima* can produce 10,000–40,000 seeds, which are easily dispersed by the wind, posing a significant risk of propagation to surrounding areas [3,4]. Additionally, the weed can propagate vegetatively through an underground rhizome that can grow to at least 20 cm in length [5,6]. Hence, exposure to exotic weeds may reduce the productivity and quality of forage crops [7,8].

*Trifolium repens* L. (Common name; white clover, family; *Fabaceae*) has a long history of therapeutic applications, owing to its diverse nutrient and mineral composition, alongside its notable nutritional, ecological, and medicinal properties. Furthermore, this forage, demonstrating heightened palatability among herbivorous livestock, is significant for its protein content [9]. The weed is cultivated for grazing, pasture, and hay, contributing directly to the wool, dairy, and meat industries, along with high-value agricultural production [10]. Additionally, it covers the soil surface and develops roots at each node, preventing soil and nutrient loss [11]. *T. repens* exhibits a strong innate capacity for heavy metal accumulation by enhancing soil enzyme activities and the microbiome, making it a valuable candidate for phytoremediation processes [12]. The invasion of non-native weeds, such as *S. altissima*, poses a potential threat to the effective cultivation of forage crops, including *T. repens*. Therefore, identifying the allelopathic effects and allelochemicals in *S. altissima* may offer novel insights into the interactions between weeds and forage crops.

Allelopathy is defined as the ability of some plant species to release chemicals into their surroundings, which can have beneficial or harmful effects on other plants [13,14]. The allelopathic potential of *S. altissima* has been shown to be mediated by polyacetylenes [15], diterpenes [16,17], flavonoid glycosides [18], and phenolic compounds [19]. Previous studies have demonstrated that biochemical analyses of *S. altissima* revealed several compounds, including polyacetylenes, triterpenes, flavonoid glycosides, phenolic compounds, monoterpenes, and sesquiterpenes. Nishidono and Tanaka [20] identified the predominant compounds in *S. altissima* as the polyacetylene (2Z, 8Z)-10-angeloyloxy matricaria ester and the diterpene 13E-kolavenic acid. Additionally, the study demonstrated that these compounds, derived from the underground parts of *S. altissima*, exhibit allelopathic effects, with cis-dehydromatricaria ester being the primary active component.

Understanding the signaling mechanisms of crops, involving the interaction of endogenous phytohormones such as abscisic acid (ABA), salicylic acid (SA), and jasmonic acid (JA) with antioxidants, is crucial for enhancing plant immunity against external stressors [21,22]. The allelopathic effects of a crop can significantly influence the cross-talk within the biochemical pathways of the host [23]. Endogenous phytohormones and antioxidants play a crucial role in scavenging toxic radicals, such as reactive oxygen species (ROS), and in maintaining osmotic balance in crops [24]. The invasion of *S. altissima* in grasslands poses a significant threat to forage production and other crops, along with wetland ecosystems. Additionally, the management of other seasonal weeds in agriculture fields hinders efficient nutrient management and crop production.

Therefore, this study aims to investigate whether allelochemicals extracted from *S. altissima* can act as bioherbicides to prevent weed growth and identify the compounds responsible for inducing allelopathy and/or phytotoxicity in forage crops. The *S. altissima* extract was treated to *T. repens* to observe its allelopathic effects, along with several WS considered potential threats to agricultural production, including *Echinochloa oryzicola* L., *Cyperus microiria* L. [25], *Alopecurus aequalis* L. [26], *Portulaca oleracea* L. [27], and *Amaranthus retroflexus* L. [28], to assess its herbicidal potential. The potential cause of this phytotoxic effect was elucidated through the identification of various allelochemicals in the *S. altissima* extract. The findings of this study could offer insights into both the benefits and risks of *S. altissima* in relation to crop production and bioherbicide development.

## 2. Results

### 2.1. Allelopathic Effects of Solidago altissima on Trifolium repens

#### 2.1.1. Effect of *S. altissima* Extracts on Seedling Fresh Weight

**Figure 1:**
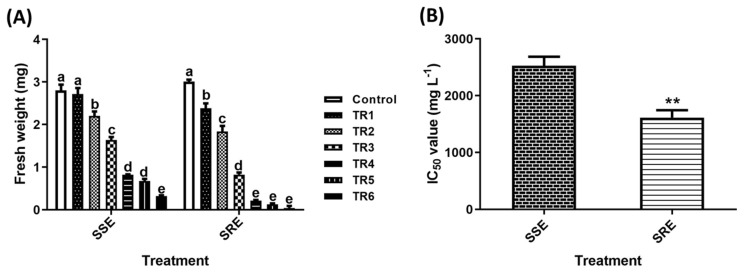
(**A**) Screening of the effects of SSE and SRE extracts on *T. repens* seedlings. (**B**) IC_50_ values of SSE and SRE extracts. The bars represent the mean ± standard deviation (SD) (n = 3). Significant differences among treatments are indicated by different letters (a–e) based on Duncan’s multiple range test (DMRT, *p* < 0.05). The IC_50_ value for each treatment was calculated based on the fresh weight of *T. repens* seedlings. Statistical significance was determined using a *t*-test (** *p* < 0.01).

In this study, the *S. altissima* seedling extract (SSE) and seed root extract (SRE) were treated with different concentrations, as described in the experimental design. The results showed a significant reduction in the fresh weight of *T. repens* seedlings, with decreases of 20%, 38%, 72%, 92%, 95%, and 98% for SRE and 3%, 26%, 45%, 72%, 77%, and 89%) for SSE at concentrations of 625, 1250, 2500, 5000, 10,000, 20,000 mg/L, respectively, compared to the control (Figure 1A). Based on these results and the IC_50_ value, the SRE extract was selected for further application in *T. repens* and other WS (Figure 1B).

#### 2.1.2. Effect of *Solidago altissima* Root Extract Foliar Treatment on the Growth and Chlorophyll Content of *Trifolium repens*

**Figure 2:**
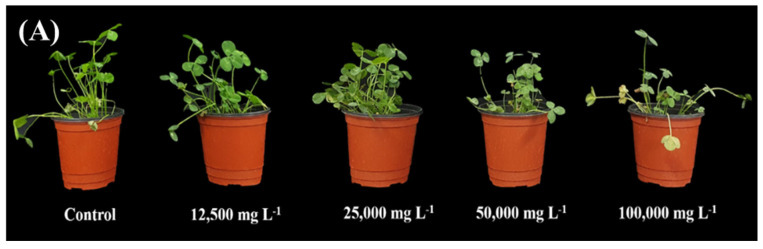

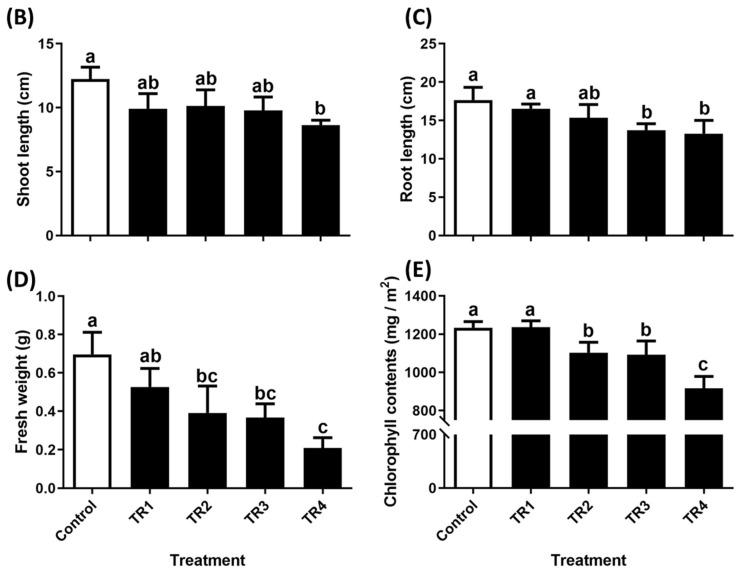
Dose-dependent effects of SRE on morphological variations in *T. repens* (**A**) Visual differences. (**B**) Shoot length. (**C**) Root length. (**D**) Fresh weight. (**E**) Chlorophyll content. Error bars represent the mean ± standard deviation (SD) (n = 5). Letters a–c denote significant differences (*p* < 0.05) using Duncan’s multiple range test (DMRT).

Subsequently, the effects of SRE foliar treatment on *T. repens* seedlings were evaluated after germination. Figure 2A illustrates the visual observations revealing a significant decline in *T. repens* appearance, with stunted plant growth as the SRE treatment increased. The SRE treatments TR2, TR3, TR4, and TR5 reduced the fresh weight by 24%, 43%, 47%, and 70%, respectively; the shoot length by 19%, 17%, 20%, and 30%; the root length by 12%, 24%, 31%, and 35%; and the chlorophyll content by 10%, 10%, 11%, and 25%, respectively, compared to that of the control (Figure 2B–E).

### 2.2. Effects of Solidago altissima Extract Foliar Treatment on Reactive Oxygen Species Content and Antioxidant Enzyme Activity

#### 2.2.1. Reactive Oxygen Species Content

**Figure 3:**
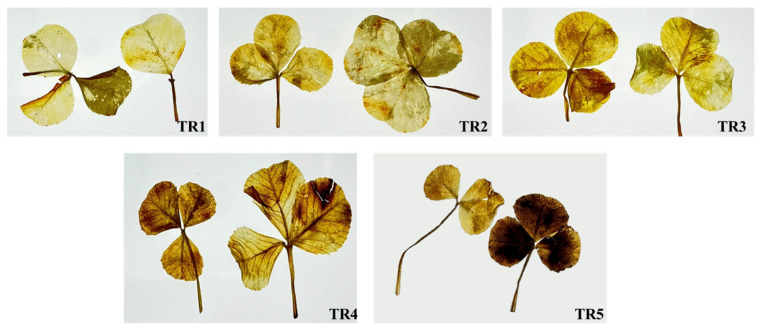
Effect of SRE treatment on H_2_O_2_ accumulation in *T. repens* seedlings, visualized using DAB staining. H_2_O_2_ concentrations in *T.repens* seedlings increased in the following order: TR5 > TR4 > TR3 > TR2 > TR1.

**Figure 4:**
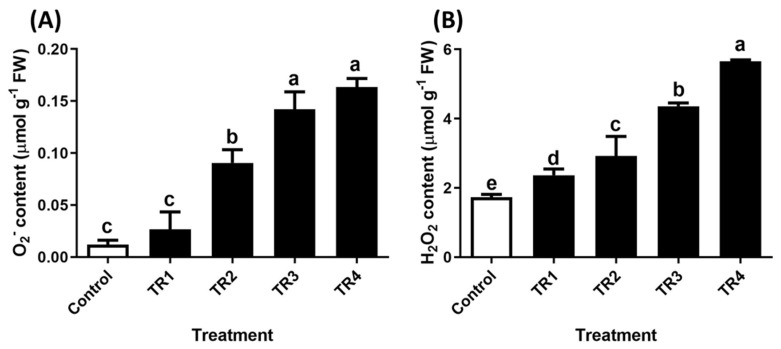
Reactive oxygen species (ROS) content in *T. repens* seedlings after SRE treatment. (**A**) O_2_^−^ content. (**B**) H_2_O_2_ content. Bars and error bars represent the mean ± standard deviation (SD), (n = 3). Different letters denote significant differences (*p* < 0.05), as determined using Duncan’s Multiple Range Test (DMRT).

The hydrogen peroxide (H_2_O_2_) content in *T. repens* seedlings was visualized using the 3,3′-Diaminobenzidine (DAB) staining method. Figure 3 and Figure 4 display the quantification results that further validate these findings. The superoxide anion (O_2_^−^) content increased by 1.2-fold, 6.6-fold, 11-fold, and 12.7-fold, while H_2_O_2_ content increased by 36%, 69%, 152%, and 227% following subsequent treatment with TR2, TR3, TR4, and TR5, respectively, compared to that of the control.

#### 2.2.2. Effects of *Solidago altissima* Root Extract Foliar Application on Antioxidant Enzyme Activity

**Figure 5:**
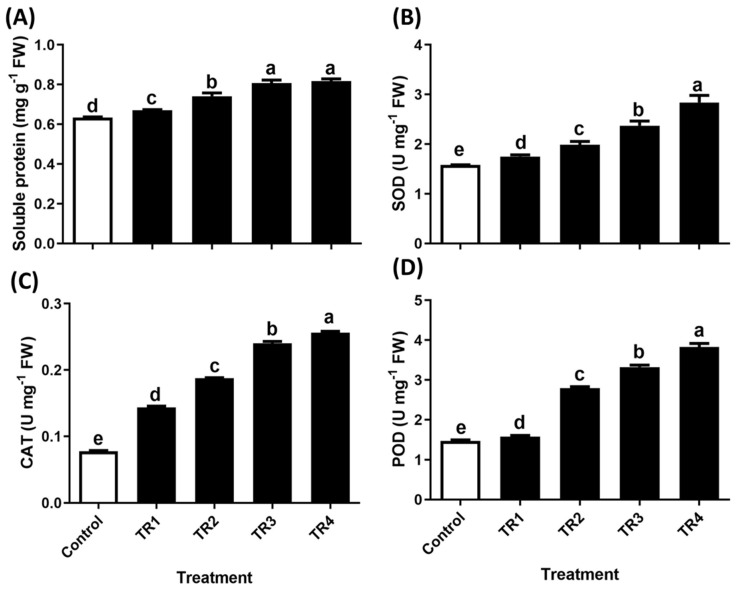
Effect of *S. altissima* extract treatment on soluble protein content and antioxidant activity in *T. repens*. (**A**) Soluble protein, (**B**) superoxide dismutase (SOD), (**C**) catalase (CAT), and (**D**) peroxidase (POD) activity. Bars and error bars represent the mean ± standard deviation (SD), (n = 3). Letters above the bars denote significant differences (*p* < 0.05), determined using Duncan’s Multiple Range Test (DMRT).

The antioxidant analysis of *T. repens* seedlings revealed that soluble protein levels increased by 6%, 17%, 27%, and 29%, superoxide dismutase (SOD) activity increased by 10%, 25%, 49%, and 79%, catalase (CAT) activity increased by 85%, 141%, 209%, and 229%, and peroxidase (POD) activity increased by 7%, 89%, 126%, and 160% following subsequent treatments with TR2, TR3, TR4, and TR5, respectively, compared to that of the control (Figure 5A–D).

### 2.3. Quantification of Endogenous Phytohormones in Trifolium repens Leaves

**Figure 6:**
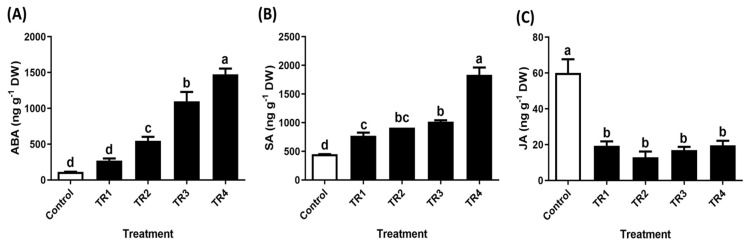
Effect of *S. altissima* extract treatment on phytohormone content in *T. repens*. (**A**) ABA, (**B**) SA, and (**C**) JA. Bars and error bars represent the mean ± standard deviation (SD), (n = 3). Letters denote significant differences (*p* < 0.05) determined using Duncan’s multiple range test (DMRT).

ABA and SA levels significantly increased with higher SRE treatment concentrations, while JA synthesis was significantly inhibited. Briefly, ABA content increased by 1.3-fold, 3.7-fold, 8.6-fold, and 12-fold, while SA content increased by 0.7-fold, 10-fold, 12.6-fold, and 3.06-fold. However, JA levels decreased by 5.7%, 17%, 27%, and 29% with increasing concentrations of TR2, TR3, TR4, and TR5, respectively, compared to that of the control (Figure 6).

### 2.4. Identification of Allelochemicals in Solidago altissima Roots

**Figure 7:**
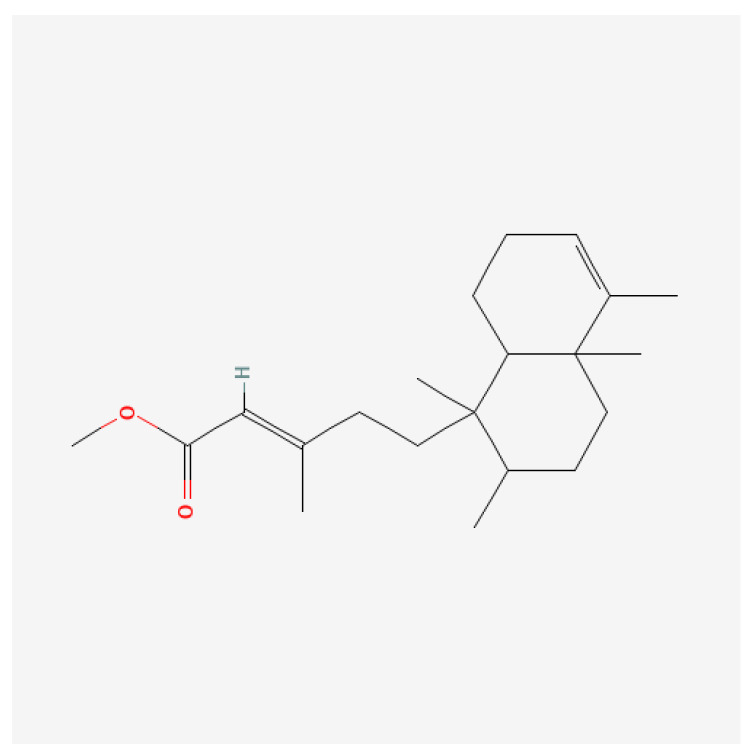
Chemical structure of the predominant compound, methyl kolavenate, identified in the extract.

Gas chromatography-mass spectrometry (GC-MS) analysis of fraction AE from the SRE extract revealed several allelochemicals. Among these, methyl kolavenate (kolavenic acid) emerged as the most abundant (35.68%) compound (Figure 7). Other compounds identified in significant quantities included n-hexadecanoic acid (4.21%), chondrillasterol (3.49%), 1,6-dibromohexane (2.76%), kolavenol (2.30%), and (E)-4,4-dimethyl-2-pentene (2.19%) (Table 1).

### 2.5. Effect of SRE Treatment on Germination Inhibition of Weed Species

**Figure 8:**
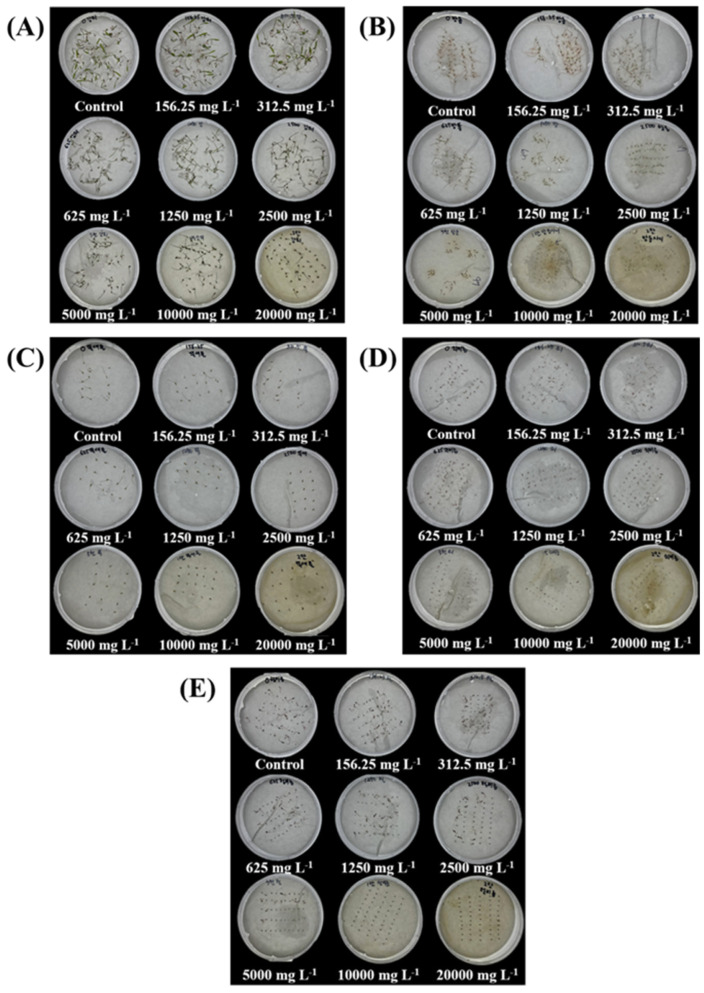
Effect of SRE treatment on the germination of different weed species: (**A**) *E. oryzicola*, (**B**) *C. microiria*, (**C**) *A. aequalis*, (**D**) *P. oleracea*, and (**E**) *A. retroflexus*.

Figure 8 illustrates the effect of different SRE concentrations on the germination of *E. oryzicola*, *C. microiria*, *A. aequalis*, *P. oleracea*, and *A. retroflexus*, with germination completely inhibited as the SRE concentration increased.

## 3. Discussion

The development of bioherbicides using extracts from invasive WS offers a promising strategy for controlling these species in agricultural settings [29]. In this study, SRE was identified as a potential bioherbicide owing to its inhibitory effects on the germination and development of *E. oryzicola*, *C. microiria*, *A. aequalis*, *P. oleracea*, and *A. retroflexus*. The phytotoxic effects of SRE treatment on *T. repens* seedlings were evaluated based on morphological levels, antioxidant activity, endogenous phytohormone levels, ROS accumulation, and allelochemical identification.

This study demonstrated that SRE treatment induced severe toxicity in *T. repens* seedlings, resulting in stunted growth, high ROS levels, and mortality at higher concentrations, indicating its potential threat to forage crop production. Furthermore, the growth of *T. repens* seedlings was significantly reduced following SRE treatment, primarily due to oxidative stress induced by ROS. The most commonly produced ROS include O_2_^−^, H_2_O_2_, hydroxyl radical (·OH), and singlet oxygen (^1^O_2_) [30]. To assess H_2_O_2_ accumulation in plants treated with SRE foliar DAB staining was conducted, revealing a significant increase, particularly at the highest SRE concentration (Figure 5). These findings are consistent with those of Kim, et al. [31], which demonstrate an extract from *Solanum carolinense* containing solanidan-3-ol (3 β,5 α) induced significant phytotoxicity in the forage crop *Festuca arundinacea* by increasing O_2_^−^ and H_2_O_2_ levels.

The identification of allelochemicals in the extract offers insights into the causative agents responsible for inducing phytotoxicity. The SRE extract was found to contain the highest proportion of the compound methyl kolavenate (35.6%). A previous study by Morimoto [32] reports that methyl kolavenate, a clerodane diterpene, exhibits antifeedant properties. Clerodane diterpenes exhibit antifeedant [33], antifungal [34], and antibacterial [35] activities. Zhao, et al. [36] suggest that methyl kolavenate and its analogs exhibit cytotoxicity, which is due to hydroxy and carboxyl functional groups. Siddiqui, et al. [37] report that the carboxyl group specifically contributes to the toxicity observed in crops. Based on these findings, methyl kolavenate present in SRE is predicted to be the primary causal agent responsible for the phytotoxic effects observed in *T. repens* and other weeds. Similar findings were reported by Barbosa, et al. [38] and Faizi, et al. [39], who demonstrates the antifeedant, antibacterial, and antifungal activities of methyl kolavenate. However, other compounds present in lower proportions in the SRE extract may contribute to its phytotoxic effects.

Allelochemicals can induce excessive ROS production, leading to the activation of free radicals and oxidizing enzymes in host plants [40]. In response, plants initiate immune defense mechanisms to detoxify ROS and mitigate damage under diverse abiotic stress conditions [41]. Our results demonstrated that SRE treatment significantly increased O_2_^−^ and H_2_O_2_ levels. Antioxidant enzymes, such as SOD, CAT, and POD, play a critical role in scavenging ROS and preventing cell death. This finding is consistent with that of Farooq, et al. [42] and Sharma, et al. [43]. The interaction among SOD, POD, and CAT is essential for the conversion of O_2_^−^ into H_2_O_2_ and subsequently into H_2_O [44,45]. In this study, CAT and POD activities were significantly increased in *T. repens* following SRE treatment. POD catalyzes the decomposition of H_2_O_2_ into H_2_O and O_2_ by oxidizing phenolic compounds or other antioxidants. In contrast, CAT removes H_2_O_2_ directly without the participation of other cofactors [46]. This finding is consistent with that of previous studies demonstrating the role of SOD, CAT, and POD in scavenging toxic radicals and enhancing stress tolerance [47,48].

The leaves of plants serve as the primary site for metabolism, where various secondary metabolites are produced and stored [49,50]. SRE treatment significantly reduced chlorophyll content, as visually observed. This was further confirmed by the quantification results, which showed a significant increase in toxic radical ions, such as O_2_^−^ and H_2_O_2._ The oxygen produced during photosynthesis accepts electrons, creating O_2_^–^ and H_2_O_2_ in photosystems I and II [51]. Considering that chlorophyll plays a crucial role in photosynthesis, particularly in the transfer and assimilation of light energy [52,53], its degradation contributes to oxidative stress.

Endogenous phytohormone modulation is a key co-factor in combating oxidative stress and maintaining crop vitality [54]. The proper synthesis of these hormones is crucial for regulating cross-signaling and modulating the biosynthetic pathways of various metabolites [55,56]. The increase in ABA and SA levels is counterbalanced by JA under external stress conditions to enhance crop growth [57,58]. Our results indicated a significant increase in ABA and SA content, while JA levels decreased in *T. repens* following foliar SRE treatment. This shift in hormone balance contributed to a significant reduction in crop growth and development, indicating higher stress levels in the plants. Overall, exogenous treatments with phytohormones, such as ABA, JA, and SA, have beneficial effects on plants under stress. However, a few studies report that these treatments can induce toxic effects. For example, Guan, et al. [59] and Refs. [58,60] demonstrate that ABA treatments in corn plants increased H_2_O_2_ and O_2_^−^ content, subsequently enhancing antioxidant enzyme synthesis.

WS management is a significant challenge in agriculture and several ecological sites. Despite the observed phytotoxic effects of SRE on *T. repens* seedlings, its application inhibited the germination and growth of all five WS, highlighting its potential as a bioherbicide.

## 4. Materials and Methods

### 4.1. Plant Material

Weed species (WS: *S. altissima*, *E. oryzicola*, *C. microiria*, *A. aequalis*, *P. oleracea*, *A. retroflexus*) were collected from different locations in Panmun-dong (Jinju-si, Republic of Korea). White clover seeds were purchased from Ubiqsolution (Guri, Republic of Korea).

### 4.2. Plant Experiment

#### 4.2.1. Preparation of *Solidago altissima* Extract

The shoots and roots of *S. altissima* were separately shade-dried and ground into fine powder. The preparation of SSE and root SRE followed the method described by Cho, et al. [61] with slight modifications. Briefly, 200 g of *S. altissima* shoot and root powder was divided into four beakers (50 g per breaker) and suspended in 500 mL of methanol (MeOH). The mixture was stirred at 500 rpm for 24 h. The resulting extracts were combined, concentrated using a rotary evaporator (Rotary Evaporator N-1100VW, Eyela, Tokyo, Japan), and reconstituted in 100 mL distilled water (d-H_2_O). The extracts were freeze-dried, yielding dried SSE and SRE, which were subsequently stored at −80 °C.

#### 4.2.2. In Vitro Seed Bioassay for *T. repens* and Weed Species

A stock solution of SSE and/or SRE at 20,000 mg/L was serially diluted with d-H_2_O to prepare different concentrations: (control; only d-H_2_O, T1; 625 mg/L), T2 1250 mg/L, T3; 2500 mg/L, T4; 5000 mg/L, T5; 10,000 mg/L, and T6; 20,000 mg/L. Two milliliters of solution from each concentration were dispensed onto a filter paper (Advantec no. 2, Toyo Roshi Kaisha Ltd., Tokyo, Japan) and placed in a Petri dish (60 × 15 mm). *T. repens* seeds were sterilized with 3% sodium hypochlorite (NaOCl) and rinsed three times with autoclaved d-H_2_O. Subsequently, 20 seeds were placed in each Petri dish and allowed to germinate for 7 days in a plant growth system (JSPC-420C, JSR Corporation, Gongju, Republic of Korea) under controlled conditions: 20 °C temperature, 60% relative humidity, 6850 lux light intensity, and 16/8 h day/night photoperiod, as described by Liu, et al. [62]. A dose-response curve was constructed based on the fresh weight of the forage crop seedlings, and the half-maximal inhibitory concentration (IC_50_) was calculated. The root extract demonstrated significant efficiency in exerting crop dominance and inducing phytotoxic effects in *T. repens*. Consequently, the root extract was selected for further analysis. Based on the results of the *T. repens* seed bioassay, the SRE was applied to WS seeds, with the stock solution further diluted to concentrations of 312.5 mg/L and 156.25 mg/L.

#### 4.2.3. *Solidago altissima* Root Treatment on *Trifolium repens* Seedlings

Overall, 20 *T. repens* seeds were sown in each pot (9 cm × 10 cm) containing a 5 cm layer of horticultural soil (Hanareum, Shinsung Mineral, Goesan, Republic of Korea) and covered with an additional 2 cm layer of soil. Several doses of SRE were prepared from a stock solution, and the treatments were designated as follows: TR1 (control, d-H_2_O only), TR2 (12,500 mg/L SRE), TR3 (25,000 mg/L SRE), TR4 (50,000 mg/L SRE), and TR5 (100,000 mgL SRE). Three weeks after sowing, 0.1% TWEEN-20 was added as a surfactant to 5 mL of each treatment solution. The experiment was conducted with three replicates, with each pot containing 20 plants. Foliar applications (spraying 5 mL/pot) were administered three times at 1-week intervals. Following the third foliar application, morphological attributes such as shoot length, root length (focused on the main root), and fresh weight were measured. Five plants exhibiting similar growth patterns were selected from each pot to assess morphological attributes. To measure fresh weight, the plants were carefully removed from the soil without drying. The chlorophyll content of *T. repens* was quantified using a portable chlorophyll content meter (CCM-300, ADC BioScientific Ltd., Herts, UK). Plant growth conditions were maintained as described in Section 4.2.2.

### 4.3. Reactive Oxygen Species Analysis in Trifolium repens

#### DAB Test and Analysis of O_2_^−^ and H_2_O_2_ Levels

For the DAB test, the leaves were excised from the plants and stained with a DAB reagent (1 mg/mL, pH 3.8) for 12 h in the dark. Subsequently, decolorization was performed using absolute ethanol (EtOH) at 85 °C. The leaves were incubated in a 60% glycerol (C_3_H_8_O_3_) solution and visualized. The methodology for quantifying O_2_^−^ was based on the approach outlined by Navari-Izzo, et al. [63]. A spectrophotometer (Multiskan GO, Thermo Scientific, Waltham, MA, USA) was used to detect O_2_^−^ and H_2_O_2_. H_2_O_2_ analysis was conducted using the OxiTec Hydrogen Peroxide/Peroxidase Assay Kit (Biomax Co., Ltd., Nowon-gul, Seoul, Republic of Korea), following the manufacturer’s instructions. Absorbance readings were taken at 580 nm and 570 nm for O_2_^−^ and H_2_O_2_, respectively.

### 4.4. Antioxidant Analysis

#### 4.4.1. Soluble Protein Content

The soluble protein content was quantified using the method described by Bradford [64] and Park, et al. [65]. Protein samples were extracted with 50 mM sodium phosphate (Na_3_PO_4_) buffer (pH 7.0) and treated with Bradford reagent (Coomassie G-250, Thermo Fisher Scientific Korea Ltd., Seoul, Republic of Korea). Absorbance reading was recorded at 595 nm using a spectrophotometer, as described in Section 4.3. A protein standard curve was generated using bovine serum albumin.

#### 4.4.2. Measurement of Superoxide Dismutase, Catalase, and Peroxidase Activities

The extraction was performed using a 50 mM phosphate buffer containing 1 mM ethylenediaminetetraacetic acid (EDTA), and 1% polyvinylpyrrolidone (PVP), and the samples were incubated at 4 °C for 10 min, followed by centrifugation (26,452× *g*, 30 min, 4 °C) to separate the supernatant. Antioxidant enzyme activity in forage was measured using commercial assay kits. The activities of SOD, CAT, and POD were analyzed using the OxiTec SOD Assay Kit, OxiTec Catalase Assay Kit, and OxiTec Hydrogen Peroxide/Peroxidase Assay Kit (Biomax Co., Ltd., Nowon-gu, Seoul, Republic of Korea), respectively, following the instructions of the manufacturer.

### 4.5. Phytohormone Analysis

#### 4.5.1. Quantification of Abscisic Acid and Jasmonic Acid

Quantification of ABA in plants was conducted following the method described by Gam, et al. [66]. A mixture of isopropanol and acetic acid (95:5) with d6-ABA as the internal standard was used for ABA extraction and calibration. Similarly, the quantification of JA in plants was performed based on the protocol described by Shahzad et al. (2016) [67]. JA was extracted using an extraction solution of acetone and citric acid (70:30), with [9,10-2H_2_]-9,10-dihydro-JA serving as the internal standard. Methylation was performed using ethereal diazomethane (CH_2_N_2_) followed by dichloromethane (CH_2_Cl_2_). The samples were analyzed using GC-MS. Quantification was determined by comparing the peak areas of ions 190 and 194 for ABA and ion 88 for JA.

#### 4.5.2. Quantification of Salicylic Acid

Endogenous SA was quantified following the method described by Seskar et al. [68]. Samples were extracted with 90% MeOH, before being dried using a Savant Speedvac concentrator (Thermo Fisher Scientific, Waltham, MA, USA). The supernatant was treated with 5% trichloroacetic acid, followed by the addition of an SA extraction solution composed of ethyl acetate (EtOAc), cyclopentane (C_5_H_10_), and–isopropanol (C_3_H_8_O) in a 49.5:49.5:1 ratio. The organic layer containing SA was dried under nitrogen (N_2_), reconstituted with 100% MeOH, and analyzed using a high-performance liquid chromatography (HPLC) system Shimadzu LC-10) equipped with an RF-10AXL detector (excitation at 305 nm, emission at 365 nm).

### 4.6. Identification of Allelochemicals in Solidago altissima

#### 4.6.1. Liquid–Liquid Extraction

To perform liquid–liquid extraction (LLE), the root powder of *S. altissima* was dissolved in d-H_2_O and transferred into a separatory funnel. Equal volumes of n-hexane (C_6_H_14_), chloroform (CHCl_3_), EtOAc, and n-butanol (BuOH) were added, each in a 1:1 ratio to d-H_2_O, to separate the solvent fractions. The solvent fractions obtained were dehydrated using anhydrous sodium sulfate (Na_2_SO_4_) and subsequently concentrated. Each concentrated organic solvent layer was freeze-dried and used in a seed bioassay, as described in Section 4.2.2.

**Figure 9:**
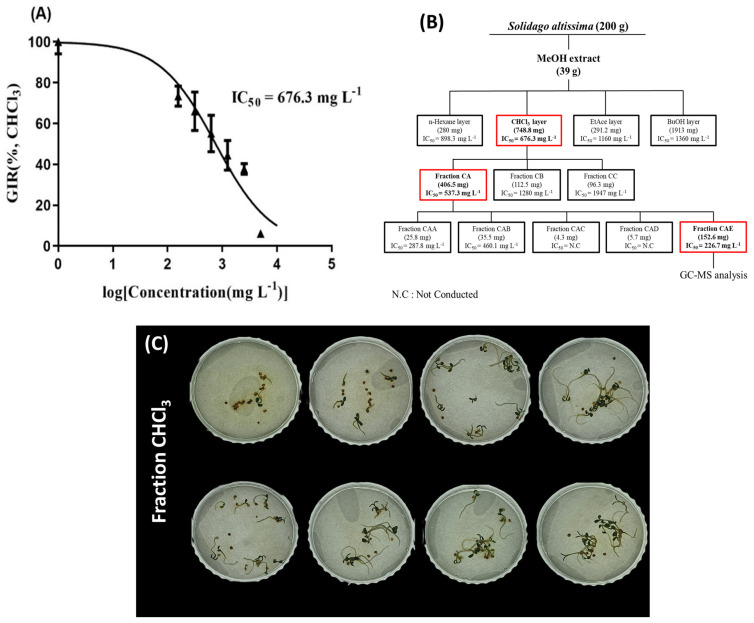
(**A**) Curve illustrating the germination inhibition rate (GIR) of *T. repens* after treatment with the CHCl_3_ fraction, with an IC_50_ of 676.3 mg/L. Error bars represent the standard deviation (SD) (n = 3). (**B**) Schematic diagram of the liquid–liquid extraction process and fraction distribution of SRE. (**C**) Visual observation of the dose-dependent effect of CHCl_3_ fraction treatment on SRE in *T. repens* germination.

Each fraction demonstrated a different IC_50_ value: C_6_H_14_ (898.3 mg/L), CHCl_3_ (676.3 mg/L), EtOAc (1160 mg/L), and BuOH (1360 mg/L). The CHCl_3_ fraction (dry weight, 748.8 mg), which exhibited the lowest IC_50_ value based on fresh weight, was selected for further separation through column chromatography. Figure 9 illustrates the LLE process diagram.

#### 4.6.2. Column Chromatography and Instrumental Analysis

The first column chromatography was performed using silica gel 60 (0.040–0.063 mm, Merck, Kenilworth, NJ, USA) with a mixed solvent system of C_6_H_14_-CHCl_3_-MeOH (5:8:1.3) as the mobile phase. Fractions CA, CB, and CC were separated, concentrated in a rotary evaporator, and subsequently used in a seed bioassay as described in Section 2.2.2. Fraction A (406.5 mg), exhibiting the lowest IC_50_ value based on fresh weight, was subjected to second-column chromatography using a mixed solvent system of C_6_H_14_-CHCl_3_ (1:10) as the mobile phase. This process yielded five fractions: CAA, CAB, CAC, CAD, and CAE. Among these, the CAE fraction, which had the lowest IC_50_, was diluted in CHCl_3_ and prepared for GC-MS analysis. The CAE fraction was analyzed in scan mode using an Agilent 7890B Network GC System combined with a 5977B Network Mass Selective Detector (Agilent Technologies, Palo Alto, CA, USA) (Table S1).

**Figure 10:**
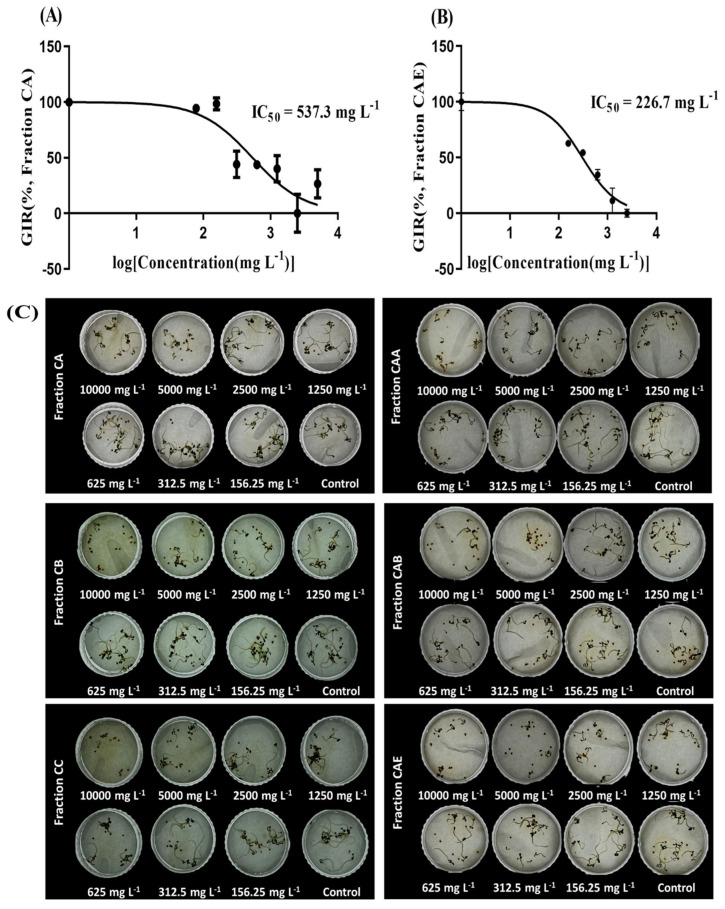
Dose-response inhibition curves illustrating germination inhibition rate (GIR) of *T. repens* for chromatography fractions with the highest IC_50_ values. (**A**) Dose-response inhibition curve of fraction CA. (**B**) Dose-response inhibition curve of fraction CAE. Error bars represent the standard deviation (SD) (n = 3). Visual observation of the dose-dependent effects of different SRE fractions on *T. repens* germination (**C**). Figure 1 details the fractionation methodology.

After primary column chromatography, three fractions—CA, CB, and CC—were collected. Each fraction was concentrated under reduced pressure, and a seed bioassay was conducted to determine their individual IC_50_ values: fraction CA (537.3 mg/L), fraction CB (1280 mg/L), and fraction CC (1947 mg/L). The second column chromatography was performed using fraction CA, which exhibited the lowest IC_50_ value, resulting in the separation of five additional fractions: CAA, CAB, CAC, CAD, and CAE. The IC_50_ values for the fractions collected in sufficient quantities for the seed bioassay were as follows: fraction CAA (287.8 mg/L), fraction CAB (460.1 mg/L), and fraction CAE (226.7 mg/L). The CAE fraction, which demonstrated the lowest IC_50_ value, was selected for GC-MS analysis. Figure 10 illustrates the germination inhibition curves for *T. repens* treated with fractions CA and AE.

### 4.7. Statistical Analysis

The in vitro seed bioassay results were analyzed using Duncan’s multiple range test (DMRT, *p* < 0.05). IC_50_ data were compared using Student’s *t*-tests (*** *p* < 0.001, ** *p* < 0.01, * *p* < 0.05). Other experimental data were analyzed using analysis of variance (ANOVA) followed by DMRT (*p* < 0.05) to identify differences among mean values. Statistical analyses were conducted using GraphPad Prism (Version 8; GraphPad Software, San Diego, CA, USA) and SAS statistical software (Version 9.4; SAS Institute, Cary, NC, USA).

## 5. Conclusions

Overall, our findings demonstrate that *S. altissima* extract exhibits allelopathic potential and contains clerodane diterpene compounds, with methyl kolavenate constituting a significant portion. This compound potentially contributes to the observed phytotoxic effects on *T. repens* by inducing oxidative stress through ROS production and germination inhibition in other weed species. To validate the specific phytotoxic effects, further experiments are required to test methyl kolavenate and other identified compounds individually across several plant species. The findings of this study offer valuable insights for developing effective strategies to manage alien invasive species and enhance forage crop production.

## Figures and Tables

**Table 1:** Major compounds identified in fraction AE extracted from *S. altissima*.

No.	Compound Name	Peak Area (%)
1	Methyl kolavenate	35.68
2	n-Hexadecanoic acid	4.21
3	Chondrillasterol	3.49
4	Carvyl angelate	2.76
5	Kolavenol	2.30
6	(E)-4,4-Dimethyl-2-pentene	2.19
7	10(E),12(Z)-Conjugated linoleic acid	1.57
8	(E)-Longipinane	1.44
9	(Z,Z)-9,12-Octadecadienoic acid	1.21

## Data Availability

Data are contained within the article and Supplementary Materials.

## Supplementary Materials

The following supporting information is available for download at https://www.mdpi.com/article/10.3390/plants14010096/s1. Figure S1: Effect of SRE organic solvent fraction treatment on the fresh weight of *T. repens* (A) C_6_H_14_, (B) CHCl_3_, (C) EtOAc, (D) BuOH. Figure S2: Effect of SRE-CHCl_3_ layer first-column chromatography fractions on the fresh weight of *T. repens* (A) fraction CA, (B) fraction CB, (C) fraction CC. Figure S3: Effect of SRE-CHCl_3_-fraction CA second-column chromatography fractions on the fresh weight of *T. repens* (A) fraction CAA, (B) fraction CAB, (C) fraction CAE. Figure S4: Effect of SRE treatment on the fresh weight of different weed species (A) *Echinochloa oryzicola*, (B) *Cyperus microiria*, (C) *Alopecurus aequalis*, (D) *Portulaca oleracea*, (E) *Amaranthus retroflexus*. Figure S5: Effect of SRE treatment on the plumule length of different weed species (A) *Echinochloa oryzicola*, (B) *Cyperus microiria*, (C) *Alopecurus aequalis*, (D) *Portulaca oleracea*, (E) *Amaranthus retroflexus.* Figure S6: Effect of SRE treatment on the radicle length of different weed species (A) *Echinochloa oryzicola*, (B) *Cyperus microiria*, (C) *Alopecurus aequalis*, (D) *Portulaca oleracea*, (E) *Amaranthus retroflexus.* Table S1: GC-MS condition for CAE fraction used in this study.

## Abbreviations

SSE	*S. altissima* shoot extract
SRE	*S. altissima* root extract
WS	Weed species
ABA	Abscisic acid
JA	Jasmonic acid
SA	Salicylic acid
ROS	Reactive oxygen species
