# Supplementary material for: Investigating the Allelopathic and Bioherbicidal Potential of Solidago altissima with a Focus on Chemical Signaling in Trifolium repens
Source: Plants (Basel). 2024 Dec 31;14(1):96. doi: 10.3390/plants14010096 (PMC11723385; doi:10.3390/plants14010096)
Supplement: Supplementary file 1 [file plants-14-00096-s001.zip › plants-3340460-supplementary.pdf]

Supplementary data

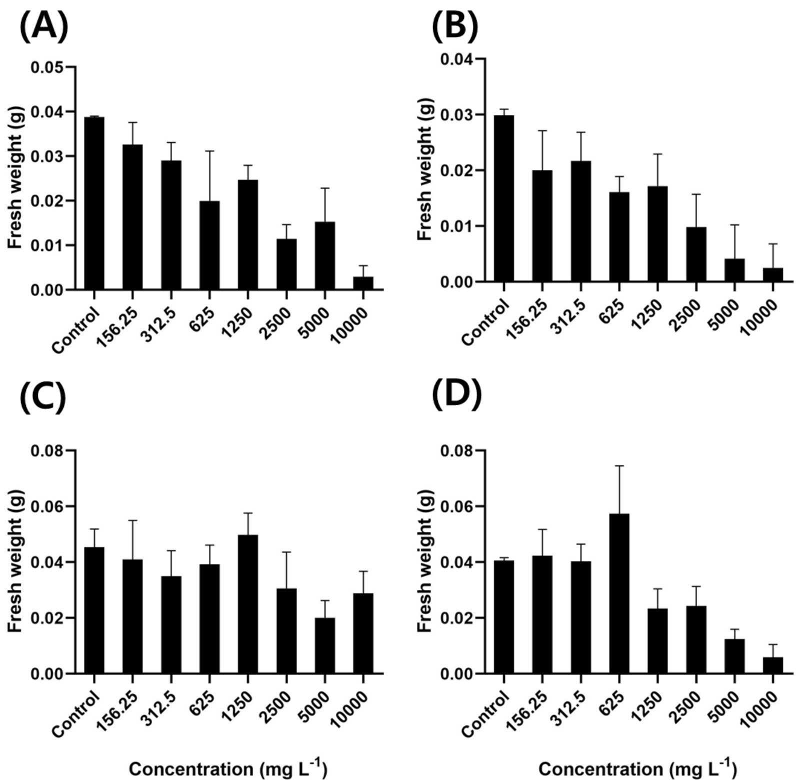

Figure S1: Effect of SRE organic solvent fraction treatment on the fresh weight of *T. repens* (A) C_6H_{14}, (B) $CHCl_3$, (C) EtOAc, (D) BuOH.

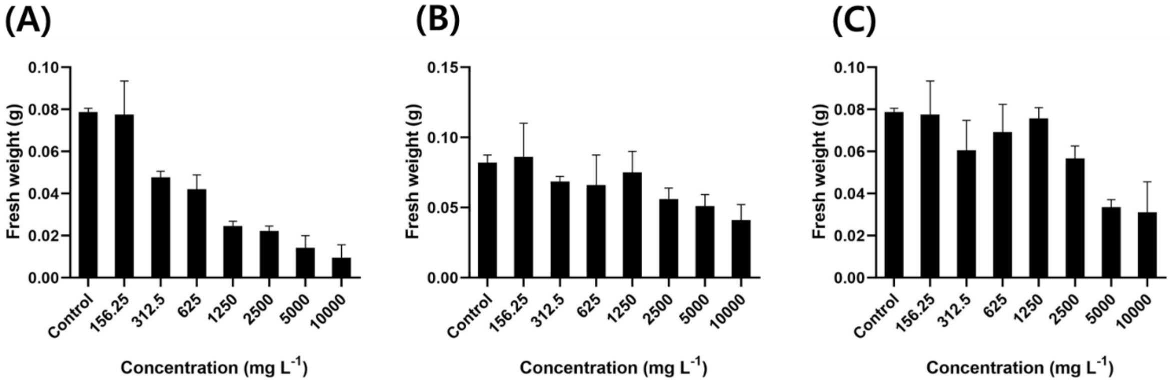

Figure S2: Effect of SRE-$CHCl_3$ layer first-column chromatography fractions on the fresh weight of *T. repens* (A) fraction CA, (B) fraction CB, (C) fraction CC.

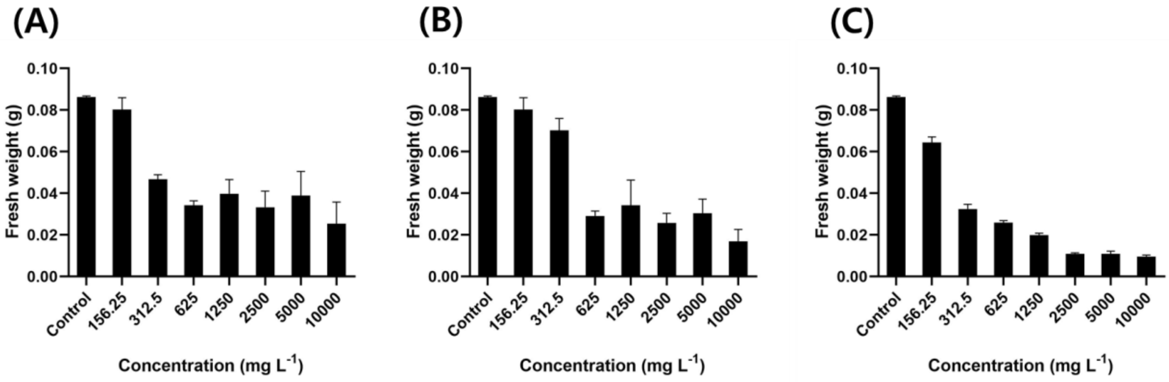

Figure S3: Effect of SRE-$CHCl_3$ -fraction CA second-column chromatography fractions on the fresh weight of *T. repens* (A) fraction CAA, (B) fraction CAB, (C) fraction CAE.

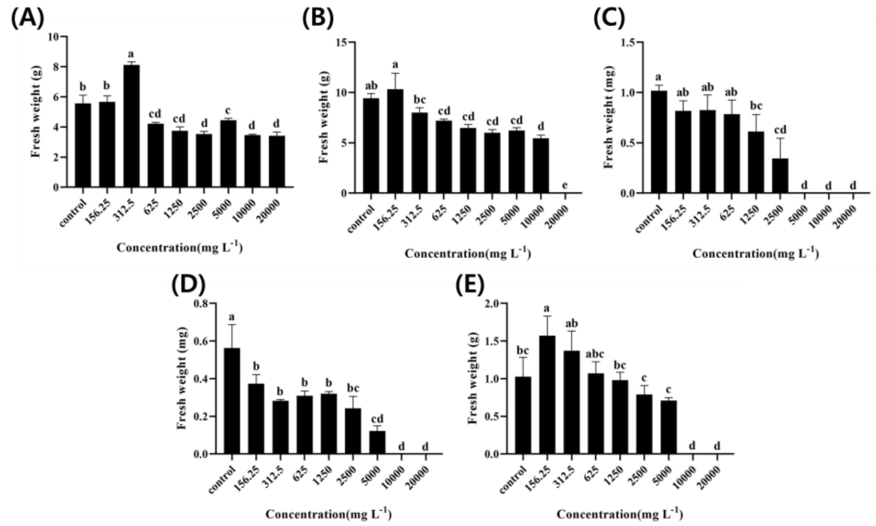

Figure S4: Effect of SRE treatment on the fresh weight of different weed species (A) *Echinochloa oryzicola,* (B) *Cyperus microiria,* (C) *Alopecurus aequalis,* (D) *Portulaca oleracea,* (E) *Amaranthus retroflexus.*

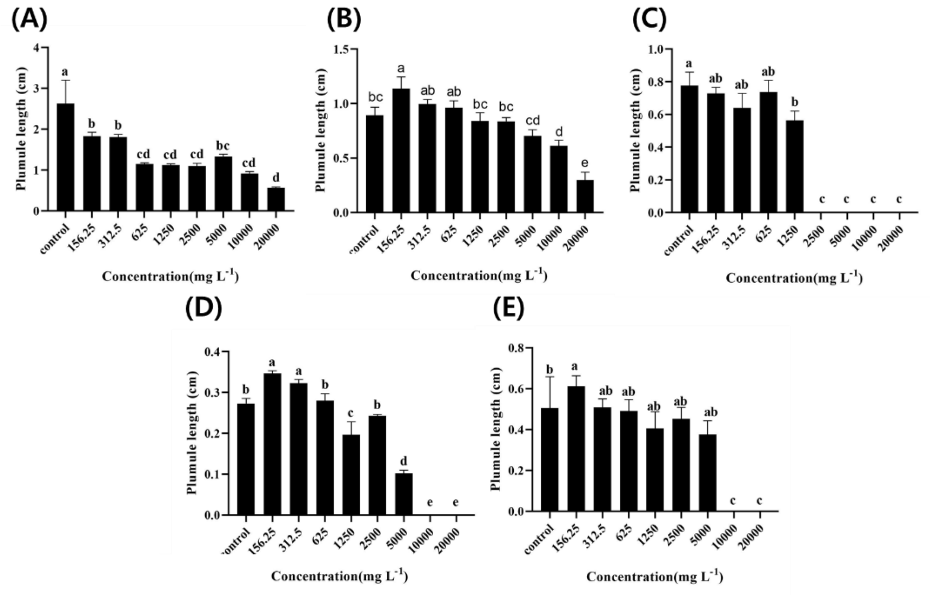

FigureS5. Effect of SRE treatment on the plumule length of different weed species (A) *Echinochloa oryzicola,* (B) *Cyperus microiria,* (C) *Alopecurus aequalis,* (D) *Portulaca oleracea,* (E) *Amaranthus retroflexus.*

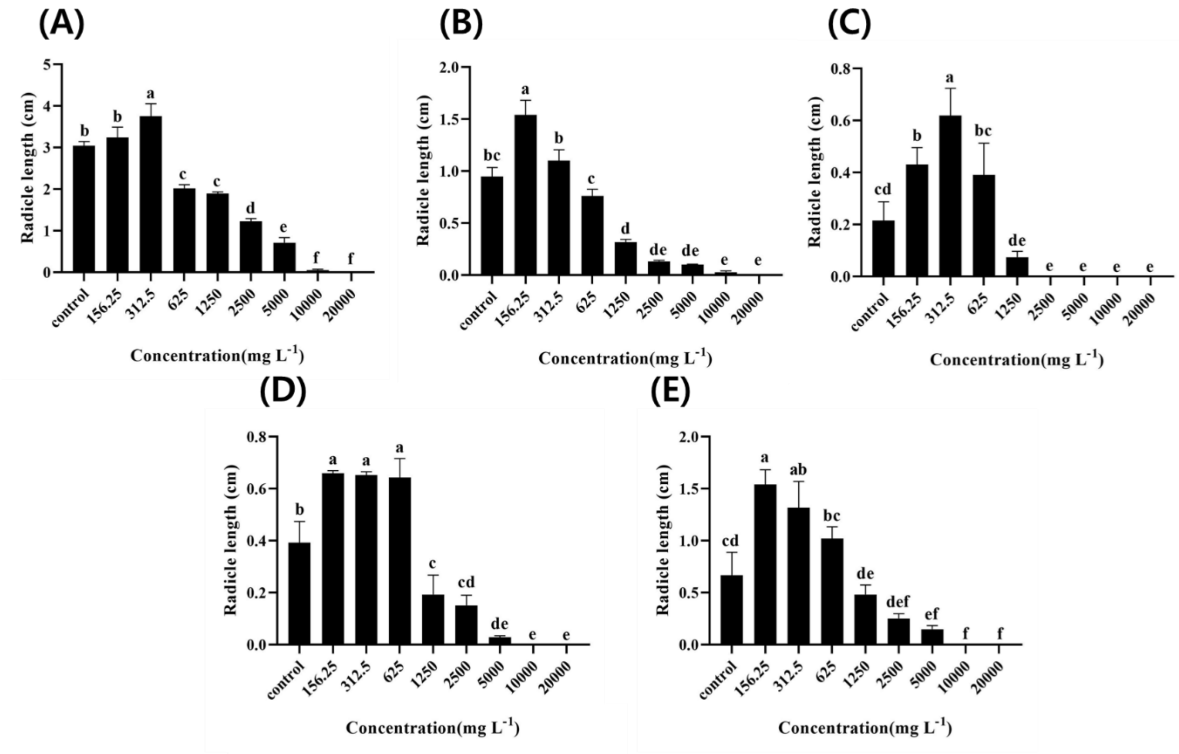

FigureS6. Effect of SRE treatment on the radicle length of different weed species (A) *Echinochloa oryzicola,* (B) *Cyperus microiria,* (C) *Alopecurus aequalis,* (D) *Portulaca oleracea,* (E) *Amaranthus retroflexus.*

TableS1: GC-MS condition for CAE fraction used in this study.

GC-MS condition for CAE fraction	
Model	Agilent 7890B
Detector	5977B Mass selective detector
Column	DB-5
Carrier gas	Helium
Ion source	Electron ionization
Ionization energy	5-241.5 eV

Appendix

Details for scientific affiliations of the plant species tested in the current experiment.

Echinochloa oryzicola (Vasinger**) Vasinger**

- **Domain:** Eukaryota
- **Kingdom:** Plantae
- **Clade:** Angiosperms
- **Clade:** Monocots
- **Order:** Poales
- **Family:** Poaceae (Grass family)
- **Subfamily:** Panicoideae
- **Tribe:** Paniceae
- **Genus:** Echinochloa
- **Species:** *Echinochloa oryzicola* (Vasinger) Vasinger

Cyperus microiria Steud. (Cyperaceae)

- **Domain:** Eukaryota
- **Kingdom:** Plantae
- **Clade:** Angiosperms
- **Clade:** Monocots
- **Order:** Poales
- **Family: Cyperaceae** (Sedge family)
- **Genus:** Cyperus
- **Species:** *Cyperus microiria* Steud.

Alopecurus aequalis Sobol. (Poaceae)

- **Domain:** Eukaryota
- **Kingdom:** Plantae

- **Clade:** Angiosperms
- **Clade:** Monocots
- **Order:** Poales
- **Family: Poaceae** (Grass family)
- **Subfamily:** Pooideae
- **Tribe:** Aveneae
- **Genus:** Alopecurus
- **Species:** *Alopecurus aequalis* Sobol.

Portulaca oleracea L. (Portulacaceae)

- **Domain:** Eukaryota
- **Kingdom:** Plantae
- **Clade:** Angiosperms
- **Clade:** Eudicots
- **Order:** Caryophyllales
- **Family:** Portulacaceae (Purslane family)
- **Genus:** Portulaca
- **Species:** *Portulaca oleracea* L.

Amaranthus retroflexus L. (Amaranthaceae)

- **Domain:** Eukaryota
- **Kingdom:** Plantae
- **Clade:** Angiosperms
- **Clade:** Eudicots
- **Order:** Caryophyllales
- **Family: Amaranthaceae** (Amaranth family)
- **Genus:** Amaranthus
- **Species:** *Amaranthus retroflexus* L.

Trifolium repens L. (Fabaceae)

- **Domain:** Eukaryota
- **Kingdom:** Plantae
- **Clade:** Angiosperms
- **Clade:** Eudicots
- **Order:** Fabales
- **Family:** Fabaceae (Legume or Pea family)
- **Subfamily:** Faboideae
- **Tribe:** Trifolieae
- **Genus:** Trifolium
- **Species:** *Trifolium repens* L.

Solidago altissima L. (Asteraceae)

- **Domain:** Eukaryota
- **Kingdom:** Plantae
- **Clade:** Angiosperms
- **Clade:** Eudicots

- **Order:** Asterales
- **Family:** Asteraceae (Aster or Sunflower family)
- **Tribe:** Astereae
- **Genus:** Solidago
- **Species:** *Solidago altissima* L.
